# Spontaneous Coronary Artery Disease (SCAD) in a Patient With Systemic Lupus Erythematosus (SLE)

**DOI:** 10.7759/cureus.43061

**Published:** 2023-08-07

**Authors:** Ravi Patel, Richa Patel, Hamfreth Rahming, Julia Tian, Ruben Kandov

**Affiliations:** 1 Internal Medicine, Northwell Health, Staten Island, USA; 2 Medicine, Baroda Medical College, Ahmedabad, IND; 3 Cardiology, Northwell Health, Staten Island, USA; 4 Interventional Cardiology, Northwell Health, Staten Island, USA

**Keywords:** non-st segment elevation myocardial infarction (nstemi), spontaneous coronary dissection, systematic lupus erythematoses, chest pain, vasculitis, lupus, sle, coronary artery dissection, scad

## Abstract

Spontaneous coronary artery dissection (SCAD) is a rare phenomenon that emerges as an acute coronary syndrome (ACS) and sudden cardiac death, especially in young women. We report a case of a woman with systemic lupus erythematosus (SLE) who presented with syncope and was found to have SCAD.

## Introduction

Spontaneous coronary artery dissection (SCAD) is defined as an epicardial coronary artery dissection that is not associated with atherosclerosis, trauma, or iatrogenic factors [1]. Cardiovascular disease due to systemic lupus erythematosus (SLE) remains one of the leading causes of death, mostly due to pericardial involvement. SLE increases the risk of premature atherosclerosis [2]. Thus, SCAD requires a different set of treatment and diagnostic workups than acute coronary syndrome (ACS), and therefore early diagnosis is essential. SCAD is rarely associated with systemic inflammatory diseases such as SLE, inflammatory bowel disease, sarcoidosis, and systemic vasculitis. So far, only eight cases have been reported in a systematic case review performed using MEDLINE/PubMed, Scopus, and Web of Science databases in May 2019 [3]. Currently, two theories are thought to be the pathophysiology of SCAD. The first proposes that the intimal tear allows blood from the true lumen to flow through the intima and form a false lumen, leading to dissection. The second theory proposes that spontaneous hemorrhage arises from Vaso vasorum, giving rise to SCAD. Chest pain is present in up to 95% of patients with SCAD; however, nausea and vomiting are the second most common symptoms, with up to 51% prevalence [1]. We report a case of a woman in her late 60s with a past medical history of SLE who presented to the hospital with syncope and was found to have SCAD during the cardiology workup for elevated troponins.

## Case presentation

A woman in her late 60s with a past medical history of Alzheimer’s dementia, epilepsy, hyperlipidemia, diabetes mellitus, hypertension, and SLE was brought to the hospital by Emergency Medical Services (EMS) after losing consciousness. She had been walking home after shopping at a grocery store when she reported experiencing palpitations, shortness of breath, flushing, and suddenly lost consciousness. She denied experiencing lightheadedness, vertigo, headache, vision changes, neurological deficits, weakness, paresthesia or tingling, nausea, vomiting, cough, chest pain, tachycardia, abdominal pain, diarrhea, or constipation. The patient's home medication list included aspirin 81 mg once a day (for primary prevention), eslicarbazepine 600 mg once a day, topiramate 200 mg two times a day, cevimeline 30 mg three times a day, and melatonin 3 mg at bedtime as needed for insomnia. The patient's hyperlipidemia, hypertension, and diabetes were managed with diet and lifestyle modifications.

**Figure 1:**
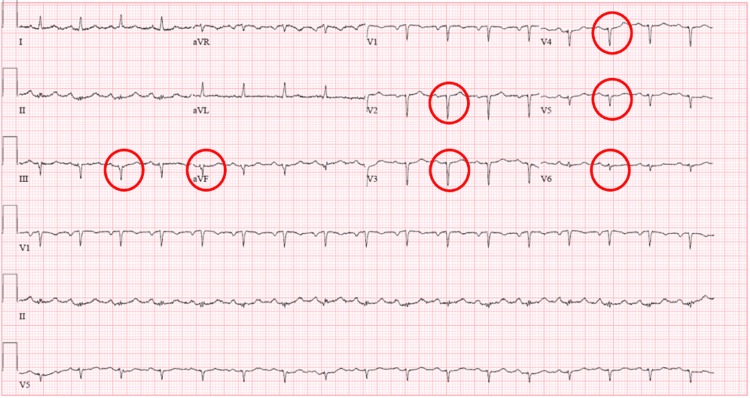
Initial electrocardiogram (EKG) showing deep Q waves in leads III, aVF, v3-v5

**Figure 2:**
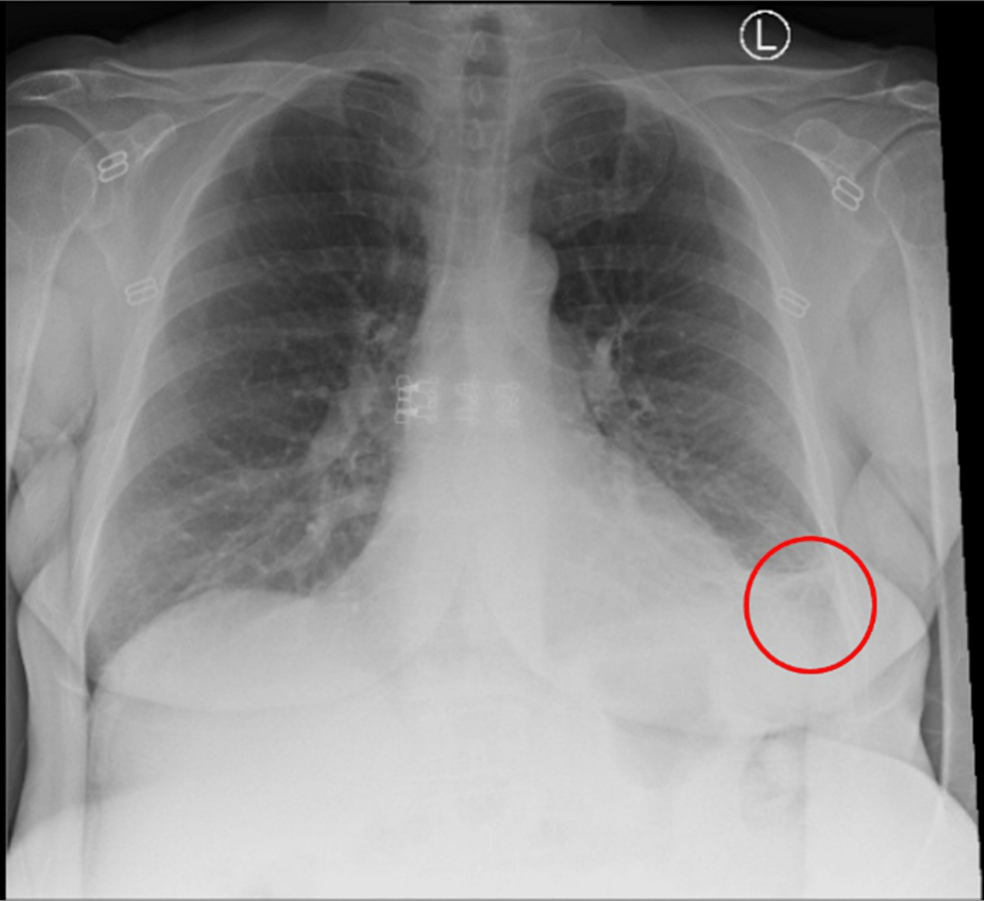
Chest x-ray showing left basilar pulmonary atelectasis vs scarring

In the emergency department (ED), the patient's vitals were stable, with a blood pressure of 116/59 mmHg, a heart rate of 127 beats per minute, a respiratory rate of 17 breaths per minute, an oral temperature of 98.6 Fahrenheit, and oxygen saturation of 95% on room air. Labs were unremarkable except for an elevated serum troponin T of 0.46 ng/mL, which peaked at 0.47 ng/mL and then downtrended to 0.27 ng/mL. Creatinine kinase was elevated to 10,604 units/liter, while creatine kinase-MB (CKMB) was elevated to 297.8 units. The electrocardiogram (EKG) showed deep Q waves in Leads III, aVF, V3-V5 (Figure 1). No prior EKGs were available for comparison. The chest x-ray showed left basilar pulmonary opacities concerning atelectasis or scarring (Figure 2). The computed tomography angiography (CTA) of the chest did not show any pulmonary embolism. The patient was admitted to the Coronary Care Unit (CCU) with a working diagnosis of non-ST elevation myocardial infarction. She was started on aspirin, clopidogrel, and a heparin drip while in the ED, pending coronary angiography.

**Figure 3:**
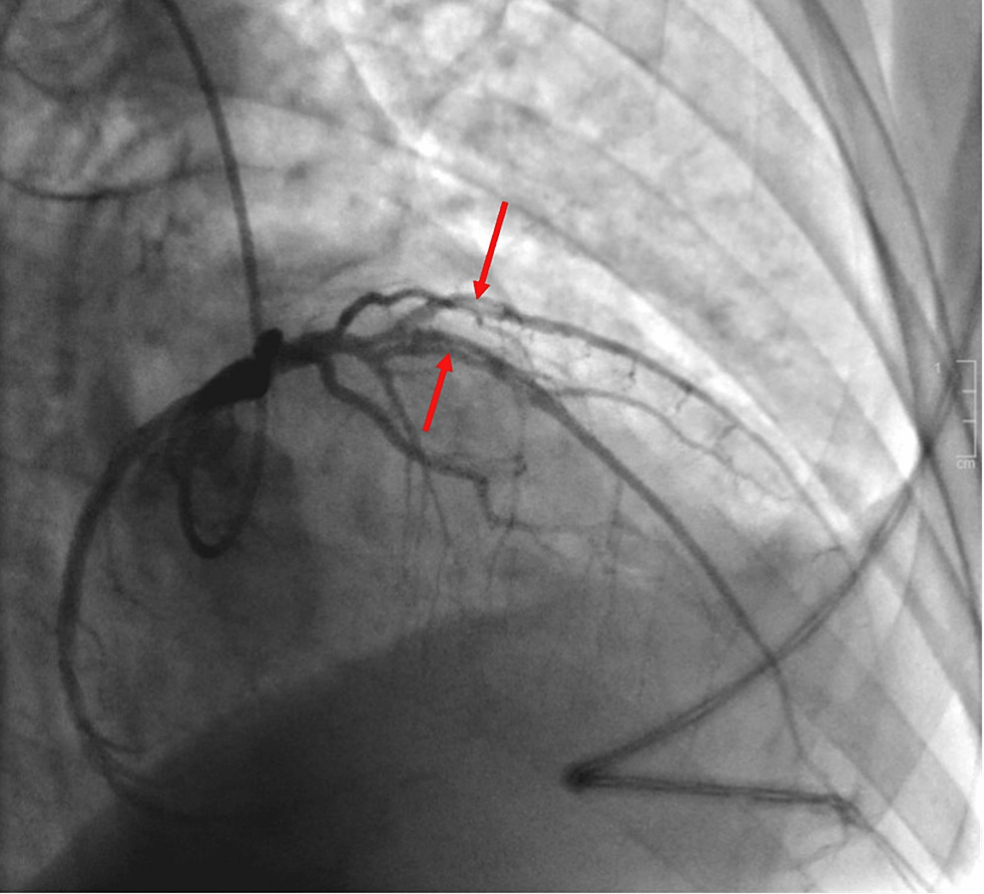
Image from cardiac catheterization with luminant red arrows showing dissection of mid-left anterior descending (LAD) artery and first diagonal artery (D1) of the LAD in the right anterior oblique - cranial view

While in the CCU, the patient underwent cardiac catheterization, which showed spontaneous coronary dissection of the mid-Left Anterior Descending (LAD) artery and first diagonal artery (D1) branch of the LAD with thrombosis in myocardial infarction (TIMI) Grade 3 flow (Figure 3). No coronary interventions were performed. Intravascular Imaging was not performed in this patient. The patient remained asymptomatic during the coronary angiogram and post-procedure. The decision was made to manage her SCAD medically.

Additionally, the patient had a CTA of the head and neck to assess any dissection in cerebral circulation or carotid circulation, both of which were unremarkable. Transthoracic echocardiogram (TTE) showed a left ventricular ejection fraction of 45%-50%, grade 1 diastolic dysfunction, mild-moderate mitral, and tricuspid valve regurgitation with mid-anterior septal, basal, and apical wall hypokinesis. No prior TTEs were available for comparison. Renal artery duplex also did not show any significant renal artery disease. Subsequent lab results showed an antinuclear factor titer of 1:320 and an anti-smooth muscle antibody titer of 1:20. The patient was discharged home on aspirin, Plavix, low-dose statin, and metoprolol succinate, with plans to follow up outpatient and increase the metoprolol dose as tolerated.

## Discussion

SCAD is defined as the separation of the coronary arterial wall due to non-traumatic or non-iatrogenic causes. Acute formation of an intramural hematoma can lead to compression of the true lumen and subsequently cause myocardial ischemia and infarction [1].

In a 2018 study by Mahmoud, 0.98% of the 752,352 women with acute myocardial infarction (AMI) were diagnosed with SCAD [4]. The true prevalence of SCAD remains uncertain as it is an underdiagnosed condition, with overall cases estimated to be between 1% and 4% of all ACS cases. Moreover, SCAD may be the cause of ACS in up to 35% of women of age 50 and below. The LAD artery and its diagonal and septal branches are affected in 45%-61% of cases. The prevalence of SCAD in pregnancy was 1.81 [1]. In a 1984 study, immunopathologic studies were conducted on extramural coronary arteries in two patients with SLE, and immune reactants were found in the walls of both inflamed and noninflamed segments, which was consistent with immune complex deposition. Neither of the two patients had evidence of significant coronary atherosclerosis [5].

Eighty percent of cases are associated with predisposing factors, including fibromuscular dysplasia (FMD), postpartum status, multiparity (≥4 births), connective tissue disorders, systemic inflammatory conditions, hormonal therapy, and may also be associated with COVID-19 inflammatory syndromes. The remaining 20% are classified under idiopathic causes [6]. Women make up 88.5% of SCAD cases, with a mean age of 51.8 +/- 10.2 years [7]. Women presenting to the hospital with AMI and SCAD are associated with higher in-hospital mortality than those presenting without SCAD (6.8% vs. 3.8%, OR: 1.87, 95% CI: 1.65 to 2.11; p < 0.0001) [4]. Additionally, SCAD is involved in 43% of ACSs in pregnant women [8]. In a recent study of 66,360 patients with SCAD, 280 were found to have SLE [9].

No routine prospective studies have performed follow-up angiographic restudy after SCAD. However, observational data show that SCAD lesions heal in most patients (70%-97%) who were selectively restudied weeks to months after conservative management. Current studies suggest that complete resolution of the dissected vessels on angiography occurs in 73%-97% of cases within four to six weeks in patients treated with conservative therapy. Revascularization with stents is associated with poor outcomes and higher failure rates due to disrupted arterial wall integrity. The passage of a guidewire may also lead to propagation of the dissection plane by entering the false lumen. Revascularization may be appropriate in patients with high-risk features such as hemodynamic instability, refractory arrhythmia, TIMI grade 0-1 flow in a proximal vessel, left main coronary artery dissection, and ongoing ischemia. However, neither percutaneous coronary intervention (PCI) nor coronary artery bypass graft (CABG) appears to be protective against recurrent dissection [1].

PCI in the setting of SCAD presenting as NSTEMI patients was found to have increased mortality (OR: 2.11, 95% CI 1.39-3.21, p < 0.0001) but not in STEMI patients (OR: 0.62, 95% CI: 0.41 to 0.96; p = 0.03) [4]. Conservative therapy includes antiplatelets such as aspirin and beta blockers as tolerated. Patients not able to tolerate beta-blockers should be offered nondihydropyridine calcium channel blockers [7]. The risk of recurrent SCAD is significantly lower Betablockers significantly lower the risk of recurrent SCAD (hazard ratio 0.36, p=0.004) [10]. Thrombolytics and anticoagulants are not currently indicated. Lipid-lowering therapy should only be initiated in patients with other risk factors that necessitate their use [7]. Angiotensin-converting enzyme inhibitors and angiotensin receptor blockers should be used only if left ventricular systolic dysfunction is present. Management of angina is also warranted using agents such as nitrates, calcium channel blockers, or ranolazine [1].

## Conclusions

Systematic inflammatory disorders are commonly associated with SCAD. Therefore, they should be considered in post-MI settings secondary to SCAD. For female patients aged 50 to 70 with NSTEMI or STEMI, we suggest a rheumatologic workup after discharge to rule out vasculitis due to a rheumatological disorder. No studies show that treating SLE immediately after SCAD in the hospital improves outcomes; therefore, outpatient assessment is preferable.
